# Treatment of Small Intestinal Bacterial Overgrowth (SIBO) in Gastrointestinal, Hepatic, Endocrine, Neurological, and Postoperative Diseases: A Comprehensive Narrative Review

**DOI:** 10.3390/medsci14020300

**Published:** 2026-06-10

**Authors:** Roman Maslennikov, Victoria Agarkova, Elena Poluektova, Anatoly Ulyanin, Oksana Zolnikova, Anastasia Kurbatova, Evgenii Kozlov, Tatyana Demina, Yury Zharikov, Alexey Sigidaev, Vladimir Ivashkin

**Affiliations:** 1 Department of Internal Medicine, Gastroenterology and Hepatology, Sechenov University, Moscow 119435, Russia; 2 Scientific and Technological Institute of Metabolic Health, Sechenov University, Moscow 119435, Russia; 3 Scientific Community for Human Microbiome Research, Moscow 119435, Russia; 4 Reference Center “Human Microbiota”, Moscow 119435, Russia; 5 Department of Clinical Immunology and Allergy, Sechenov University, Moscow 119991, Russia; 6 Department of Human Anatomy and Histology, Sechenov University, Moscow 125009, Russia; 7 Department of Clinical Disciplines, Tyumen State Medical University, Tyumen 625023, Russia

**Keywords:** gut microbiota, dysbiosis, irritable bowel syndrome, functional bloating, cirrhosis, Crohn’s disease, lactose intolerance, celiac disease, diverticular disease, chronic intestinal pseudo-obstruction, idiopathic halitosis, pancreatitis, functional dyspepsia, cystic fibrosis, idiopathic halitosis, systemic sclerosis, hypothyroidism, acromegaly, Parkinson’s disease, rosacea

## Abstract

Small intestinal bacterial overgrowth (SIBO) refers to an abnormal increase in the number of bacteria in the small intestine and is observed in various diseases. SIBO can also develop after long-term use of proton pump inhibitors (drug-induced SIBO), bariatric surgery, gastrectomy, and other surgeries (postoperative SIBO). The aim of this narrative review is to summarize all of the published information on the treatment of SIBO in as much detail as possible and present it separately for each specific disease and intervention associated with SIBO. The most extensively studied drug for the treatment of SIBO is rifaximin. It eliminates SIBO in 63% of cases; however, most studies lack a control group. Small RCTs assessing the effects of this antibiotic on SIBO have reported conflicting results, and a meta-analysis showed no effect. A large RCT is required to verify the results of uncontrolled studies. Neomycin and norfloxacin showed efficacy in the treatment of SIBO in single RCTs, with elimination rates of 20 and 100%, respectively. Ciprofloxacin, rifamycin, metronidazole, and other antibiotics, as well as ursodeoxycholic acid, showed positive effects for the treatment of SIBO, but only in uncontrolled studies or in comparison with rifaximin or other drugs. The reported elimination rates were 54%, 67%, 79%, and 75%, respectively. Eradication therapy for Helicobacter pylori infection eliminated SIBO at a rate of approximately 70%. Probiotics have been tested for treatment of SIBO in various diseases. VSL#3 and *Saccharomyces boulardii* CNCM I-745 were effective in RCTs, with elimination rates of 58% and 80%, respectively. In conclusion, when selecting SIBO treatment regimens, those that have demonstrated the greatest efficacy for a specific concomitant disease should be preferred, despite the generally low level of evidence supporting these approaches in most cases.

## 1. Introduction

Small intestinal bacterial overgrowth (SIBO) refers to an abnormal increase in the number of bacteria in the small intestine [1,2,3]. The important role of the gut and its microbiota in maintaining the functioning of the entire body was recently demonstrated. This has led to the development of the gut–brain, gut–metabolism, gut–liver [4], and other axis concepts. Therefore, SIBO should not be considered only a localized intestinal disorder, and its treatment is a pressing issue not only for gastroenterologists but also for doctors in other specialties.

### 1.1. SIBO Diagnosis

**Figure 1:**
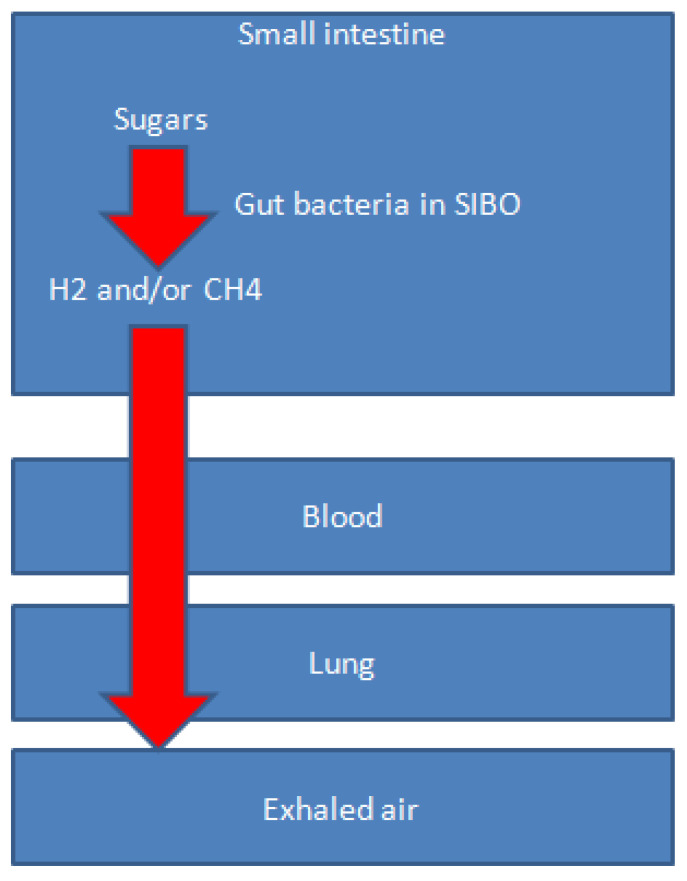
The principle of breath testing for small intestinal bacterial overgrowth.

Initially, quantitative analysis of small intestinal bacterial cultures was used for the diagnosis of SIBO; however, due to the difficulty of obtaining such cultures, this method was gradually abandoned and has been replaced with breath tests. The principle of the latter is that, in the presence of excess bacteria in the small intestine, ingested carbohydrates are metabolized by the bacteria, resulting in the formation of a large amount of gas. This gas leaves the body through various routes, including exhaled air, and can thus be determined using special analyzers (Figure 1) [5,6,7].

Historically, the first gas to be determined in this context was hydrogen, and an increase in its release after a carbohydrate load was termed hydrogen SIBO. Later, it was revealed that the small intestinal bacteria can excessively produce methane in some patients with SIBO, and this condition was termed methane SIBO. If both gases are excessively produced, then the condition is considered mixed SIBO. Hydrogen and methane SIBO have different effects on the body. In particular, hydrogen SIBO is generally characterized by diarrhea, whereas methane SIBO is characterized by a reduction in intestinal motility and constipation [8,9].

### 1.2. The Clinical Significance and Pathophysiology of SIBO

**Figure 2:**
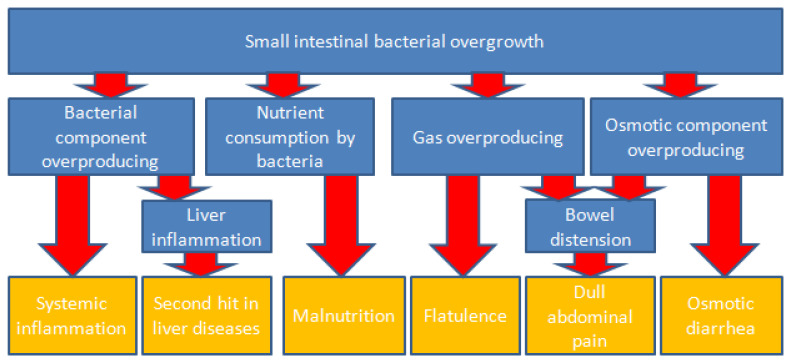
Pathogenetic significance of small intestinal bacterial overgrowth.

Gas produced by gut bacteria in the context of SIBO stretches the intestinal walls, which manifests as bloating and abdominal pain. Osmotically active substances that are also produced by these bacteria provoke the development of osmotic diarrhea, while the slowing of intestinal motility due to methane production leads to constipation. Thus, SIBO can mimic irritable bowel syndrome (IBS) and functional bloating or may contribute substantially to their symptoms [1,2,3,4]. The increased number of small intestinal bacteria facilitates their penetration into the portal bloodstream and the liver and, with a decrease in the barrier function of the latter, into the systemic bloodstream. This process is called bacterial translocation and is associated with a more severe course of metabolic-associated fatty liver disease and cirrhosis (Figure 2) [10,11]. SIBO is also associated with an unfavorable prognosis in cirrhosis [12], whereas its eradication leads to improved survival [13].

### 1.3. SIBO and Underlying Conditions

As discussed above, SIBO mimics functional bowel diseases and is detected in 33% of patients with complaints of digestive disorders [14]. It is difficult to conclude whether SIBO is a primary condition, a manifestation of functional bowel diseases, or whether it shares the same primary cause as functional bowel diseases [15,16,17].

The most common cause of drug-induced SIBO is proton pump inhibitor use, which reduces the bactericidal capabilities of the stomach and increases the flow of bacteria from the mouth into the small intestine [18]. Postoperative SIBO is most often observed as a complication of bariatric surgeries, and it is also due to impairment of the bactericidal function of the stomach [19,20].

SIBO can develop as a complication of various diseases [21]. For example, it may occur when small intestinal clearance slows down in patients with hypothyroidism [22], portal enteropathy [11], or systemic sclerosis [23]. SIBO can also develop when the supply of nutrients to the terminal sections of the small intestine increases in those with lactase deficiency [24], chronic pancreatitis [25], and other diseases [21]. In some cases, feedback occurs between SIBO and certain diseases. For example, through an increase in intra-abdominal pressure, SIBO can lead to the development of erosive gastroesophageal reflux disease [26].

Different mechanisms of SIBO development in the context of different underlying conditions can lead to different responses to therapy. For example, antibacterial drugs may be more effective in patients with normal intestinal motility and significantly less effective in patients with intestinal anatomy disruption. Prokinetic agents can lead to the resolution of SIBO in patients with slow intestinal motility but are unlikely to be effective in patients who developed SIBO while taking proton pump inhibitors.

### 1.4. Rationale for the Review

Given that SIBO significantly reduces the quality of life of affected individuals [27], causing digestive disorders [1] and aggravating the course of the many diseases associated with it [21], it is important to eradicate this pathological condition. To this end, the use of antibiotics, probiotics, and other drugs has been proposed.

Clinical guidelines provide limited information about the effectiveness of various agents in the treatment of SIBO [1,2,28,29]. Previously published reviews have generally described the effectiveness of certain drugs or groups of drugs in SIBO patients, including rifaximin, other antibiotics, or probiotics [30,31,32]. Because SIBO may have different underlying mechanisms in the context of different underlying conditions, treatment effectiveness may vary. The aim of our narrative review was to summarize all of the published information on the treatment of SIBO in as much detail as possible and present it separately for each specific disease and intervention associated with SIBO.

We used PubMed as a source and did not limit the publication time. The search term “small intestinal bacterial overgrowth” was used. In this review, we include only those studies in which the effectiveness of SIBO eradication was assessed based on a negative test for this pathological condition (usually a breath test) after therapy. Unfortunately, a number of studies did not provide such data [33], instead only reporting decreases in the level of exhaled intestinal gases or in the severity of intestinal symptoms. These studies were not included in this review, as the eradication of SIBO cannot be concluded based on these findings. The quality of the studies was assessed primarily by their design (placebo-controlled randomized controlled trial (RCT), RCT without placebo, uncontrolled study) and the number of participants. A total of over 2000 references were found. After excluding all irrelevant articles, 89 studies and 6 meta-analyses were included in this review.

## 2. Rifaximin for the Treatment of SIBO: A Summary of a Recent Meta-Analysis of Studies

The most widely used drug in the treatment of SIBO is the non-absorbable antibiotic rifaximin, and the main advantage of the use of this drug is that it has rare systemic side effects [34,35,36]. A meta-analysis of its use in the treatment of SIBO was recently published [32]. Given the significant role of this drug in the treatment of SIBO, it would be prudent to present a summary of the results of that meta-analysis before describing the treatment of SIBO in the context of specific conditions in detail. According to that meta-analysis, rifaximin eliminated SIBO in 63% of cases. This rate was 90% for SIBO with cirrhosis and approximately 60% for SIBO in the context of functional bowel diseases and Parkinson’s disease. The most commonly used dose of rifaximin was 1200 mg, with an effectiveness of 64%. The efficacy of different doses of rifaximin is presented in Table 1 [32].

The effectiveness of the therapy did not differ significantly based on duration (3, 5, 7, 10, 14, or 28 days). However, most of the studies analyzed did not include a control group that received a placebo or no treatment. When only RCTs with placebo controls were analyzed (of which there were only 4), the effectiveness of rifaximin was not superior to placebo. It should be noted that all of these RCTs were small and included fewer than 30 patients. In most studies, the elimination of SIBO resulted in a decrease in the severity of digestive symptoms. Rifaximin was not associated with adverse effects or was only associated with mild adverse effects [32]. The advantages of rifaximin include its minimal effect on the intestinal bacterial resistome [37].

## 3. Treatment of SIBO in Gut Diseases

The treatment of SIBO has been most extensively studied in gut diseases, which can be divided into functional disorders, in which there are no signs of anatomical or histological pathology (irritable bowel syndrome, functional constipation, etc.), and non-functional disorders, in which these signs are present (inflammatory bowel disease, celiac disease, etc.).

### 3.1. Treatment of SIBO in Functional Bowel Diseases

SIBO is most often found in patients with a preliminary diagnosis of functional bowel disease, including IBS, functional constipation, functional diarrhea, functional bloating, and similar diseases. SIBO has been detected in one-third of patients with IBS [16], 43% of patients with functional bloating [38], and approximately 70% of patients with functional constipation or diarrhea [39].

#### 3.1.1. Rifaximin for the Treatment of SIBO in Functional Bowel Diseases

Uncontrolled studies have shown that rifaximin resolves SIBO in 29.3–87.5% of patients with diarrhea-predominant IBS (Table 2) [40,41,42,43,44].

Rifaximin use in IBS patients improved digestive symptoms exclusively in those diagnosed with SIBO [45,46]. Treatment with 800 mg rifaximin daily for 14 days eliminated SIBO in 86% of patients, and the positive clinical effects of SIBO eradication lasted for at least 3 months [46]. A meta-analysis found that the use of antibiotics in IBS patients led to greater symptom relief when the patients had SIBO [47].

Several studies have directly compared the effectiveness of different doses of rifaximin in terms of relieving SIBO in the context of IBS. A 5-day course of rifaximin at a dose of 800 mg/day had the same effect as a 5-day course of rifaximin at 1200 mg/day [48]. When comparing 7-day courses, a dose of 1200 mg per day was preferable to 600 mg and 800 mg [49], and a dose of 1600 mg/day was preferable to 1200 mg/day [50]. Increasing the dose of rifaximin did not result in an increase in the incidence of adverse effects [50]. An indirect comparison revealed approximately equal effectiveness of 7- and 14-day courses of rifaximin at a dose of 1200 mg [51,52].

Rifaximin at 1200 mg/day for 14 days followed by a 20-day cycle of probiotics allowed for the stable eradication of SIBO in 82.6% of patients with IBS, and this effect persisted for 4–5 months [53]. However, in another study, the stability of SIBO eradication over 3–6 months was demonstrated in 86.7% of patients receiving rifaximin at a dose of 600 mg/day without any probiotics [54]. Rifaximin at 800 mg for 28 days eliminated hydrogen and methane SIBO with equal effectiveness (54.5% vs. 50%) [55]. The antibacterial drug ciprofloxacin at 500 mg/d for 7 days was ineffective in patients with SIBO who did not respond to rifaximin, eliminating SIBO in only 8% of these patients [56].

Rifaximin at 600 mg daily for 1 week eliminated SIBO in 66% of children with IBS, and the effect was accompanied by an improvement in digestive symptoms [57].

A randomized study without a placebo group showed that the addition of partially hydrolyzed guar gum at 5 g/day to a 10-day course of rifaximin at 1200 mg/day increased the SIBO eradication rate from 62.1% to 87.1%. In total, 87–91% of patients with eliminated SIBO also demonstrated a significant improvement in digestive symptoms. The positive effect of this dietary supplement is explained by its ability to normalize intestinal motility, the disruption of which is considered one of the main causes of SIBO [58].

Rifaximin at a dose of 1200 mg/day for 2 weeks eliminated SIBO in 36% of patients with functional bloating, whereas the addition of trimebutine (600 mg/day) had no significant effect [59]. A 10-day course of rifaximin at the same dose led to SIBO elimination in 42% of patients with this disease [60].

**Table 2:** The efficacy of rifaximin in the treatment of SIBO in functional bowel diseases according to uncontrolled studies.

Disease	Dose	SIBO Elimination Rate	Ref.
Diarrhea-predominant IBS	800 mg for 14 days	44–64%	[40,41]
	800 mg for 30 days	75%	[42]
	1200 mg for 14 days	87.5%	[43]
	1650 mg for 14 days	29.3%	[44]
IBS	1200 mg for 7 days	50%	[45]
	800 mg for 14 days	86%	[46]
	800 mg for 5 days	67%	[48]
	1200 mg for 5 days	67%	[48]
	600 mg for 7 days	16.7%	[49]
	800 mg for 7 days	26.7%	[49]
	1200 mg for 7 days	60%	[49]
	1200 mg for 7 days	61%	[50]
	1600 mg for 7 days	82%	[50]
	1200 mg for 7 days	85.5%	[51]
	1200 mg for 14 days	85.7%	[52]
	600 mg for 10 days	86.7%	[55]
	800 mg for 28 days	50–55%	[55]
IBS in children	600 mg for 7 days	66%	[57]
Functional bloating	1200 mg for 14 days	36%	[59]
	1200 mg for 10 days	42%	[60]

Note: Studies with complex designs are not included.

SIBO recurred at 3, 6, and 9 months after successful eradication with rifaximin (at a dose of 1200 mg/day for 10 days) in 12.6%, 27.5%, and 43.7% of patients, respectively. Predictors of recurrence were a history of appendectomy, use of proton pump inhibitors, and older age. SIBO recurrence was associated with the return of digestive symptoms [61].

Therefore, most uncontrolled studies have shown that rifaximin is effective in the treatment of SIBO in the context of functional bowel diseases; however, the specific eradication rates varied.

#### 3.1.2. Other Antibacterial Drugs for the Treatment of SIBO in Functional Bowel Diseases

The effectiveness of other antibacterial drugs in the treatment of SIBO has also been studied (Table 3).

Metronidazole or ornidazole at 1500 mg per day for 10 days per month for 3 months led to the elimination of SIBO in 52% of patients. In addition, norfloxacine at 800 mg per day, ciprofloxacin at 1000 mg per day, or ofloxacin at 400 mg per day for 10 consecutive days per month for 3 months eliminated SIBO in 50% of patients. When these (quinolone and azole) regimens were alternated monthly for 3 months, the effectiveness of the therapy increased to 70% [62].

**Table 3:** The efficacy of antibiotics other than rifaximin in the treatment of SIBO in functional bowel diseases.

Drug	Dose	SIBO Elimination Rate	Ref.
Rifamycin	776 mg for 14 days	67%/40% *	[63]
	1164 mg for 14 days	46%/38% *	[63]
Tilichinol 400 mg + tilbroquinol 800 mg for 10 days		52.5%	[64]
Trimethoprim–sulfamethoxazole 30 mg/kg + metronidazole 20 mg/kg for 14 days		95% **	[65]
Colimycin 405 mg + gentamycin 300 mg for 10 days		42%	[66]
Chlortetracycline	1000 mg for 7 days	27% ***	[67]
Metronidazole	750 mg for 7 days	43.7% ***	[68]
	1500 mg for 10 days	79% ***	[69]
Ciprofloxacin	1000 mg for 10 days	54% ***	[69]
Neomycin	1000 mg for 7 days	20% ****	[70]
Norfloxacin	800 mg for 10 days	100% ****^/^*****	[71]

Note: * Hydrogen/methane SIBO, ** in children, *** randomized study without placebo, **** randomized study with placebo, ***** very small study. Studies with complex designs are not included.

Rifamycin (388 mg twice a day vs. 388 mg three times a day) for 14 days eradicated hydrogen SIBO in 67% and 46% of cases and methane SIBO in 40% and 38% of cases, respectively. In the first group of patients, the improvement in breath test results coincided with improvements in abdominal pain, bloating, belching, and flatulence in 25% of patients. In the second group, these improvements were observed in 53% of patients. The severity of constipation generally remained unchanged in the first group of patients but decreased in the second group [63].

Khalighi et al. conducted a comparative analysis on the effectiveness of maintenance therapy using minocycline (100 mg twice a day) with Bacillus coagulans spores and fructo-oligosaccharides for 15 days every month compared with minocycline alone. Before the use of these regimens, an unspecified “aggressive therapy with broad-spectrum antibiotics” was administered. The maintenance therapy itself lasted 6 months. In the antibiotic + symbiotic group, 93.3% of patients did not have SIBO after 6 months of maintenance therapy. In the antibiotic without symbiotic group, only 67.7% achieved this result [72].

Tilichinol (400 mg/day) and tilbroquinol (800 mg/day) for 10 days eliminated SIBO in 52.5% of patients [64], whereas the use of trimethoprim–sulfamethoxazole (30 mg/kg per day) and metronidazole (20 mg/kg per day) for 14 days eliminated SIBO in 95% of children [65]. The combination of colimycin (405 mg/day) and gentamycin (300 mg/day) for 10 days eliminated SIBO in 42% of patients with functional bowel diseases. The frequency of SIBO eradication with respect to specific forms of these diseases (i.e., IBS with constipation, IBS with diarrhea, mixed and unspecified IBS, functional constipation, functional bloating, functional diarrhea, functional abdominal pain) did not differ significantly, with eradication rates of approximately 40% noted in the context of all of these conditions [66].

Attar et al. published a study with a rather complicated design. After a period of taking a placebo (which had no effect on SIBO), 10 patients were randomized to norfloxacin (800 mg/day), amoxicillin–clavulanic acid (1500 mg/day), and *Saccharomyces boulardii* (1500 mg/day) for 7 days. Patients in whom SIBO did not resolve were given one of the other drugs. In the end, norfloxacin resolved SIBO in three patients, amoxicillin–clavulanic acid in five patients, and *Saccharomyces boulardii* in none. Because it was not stated how many patients received each treatment, it was not possible to assess their effectiveness [73].

Several randomized trials have compared the effectiveness of rifaximin with that of other antibiotics (Table 4). Rifaximin eradicated SIBO more effectively than chlortetracycline [67] and metronidazole (750 mg/day) [68]. A recent randomized trial compared the effects of 10-day courses of rifaximin-α (800 mg/day), ciprofloxacin (1000 mg/day), and metronidazole (1500 mg/day) on SIBO eradication in IBS patients. At this high dose, metronidazole demonstrated the best results, whereas there was no significant difference in the elimination of SIBO between rifaximin and ciprofloxacin. However, rifaximin caused fewer side effects (9%) compared with the other antibiotics (approximately 40% for each) [69].

Therefore, antibiotics other than rifaximin were also able to eradicate SIBO. In some cases, the effect was comparable to rifaximin; in others, the effect was superior or inferior.

#### 3.1.3. Randomized Placebo-Controlled Trials of Antibiotics for SIBO Treatment in Functional Bowel Diseases

A major weakness of all the studies described above was the absence of a placebo or untreated control group. When classical RCTs are analyzed, the findings appear less encouraging (Table 4).

A small RCT (with 15 and 11 people with SIBO in each group) showed that rifaximin at 550 mg twice daily for 2 weeks was not more effective than placebo in eliminating SIBO in non-constipation IBS patients (13% vs. 18% for hydrogen SIBO and 14% vs. 33%, respectively) [74]. Rifaximin at 550 mg 3 times per day for 10 days was not more effective than placebo in eliminating SIBO in children with chronic abdominal pain in another RCT (20% vs. 14%) [75].

Unlike rifaximin, neomycin at 500 mg bid for 10 days eliminated SIBO in IBS more effectively than placebo (20% vs. 2%) in a large RCT (41 and 43 patients in the treatment and control groups, respectively). Patients with eliminated SIBO reported a significantly greater reduction in symptoms. No significant side effects were noted [70]. Norfloxacin at 800 mg/day for 10 days eliminated SIBO in all 4 IBS patients, which was superior to placebo (4/4 vs. 0/4; *p* = 0.014) in a very small RCT. The test was performed 1 month after the end of therapy [71].

Therefore, in contrast to uncontrolled studies and active-controlled RCTs, placebo-controlled RCTs showed significantly lower efficacy for rifaximin, which was indistinguishable from placebo. Although superior to placebo, neomycin demonstrated a very low elimination rate, and the trial with norfloxacin had a small number of participants. Larger placebo-controlled RCTs with rifaximin and other antibiotics are needed to confirm the high efficacy rates of these drugs that were previously reported in uncontrolled studies.

#### 3.1.4. Probiotics for the Treatment of SIBO in Functional Bowel Diseases

In addition to antibiotics, probiotics, which are preparations containing live bacteria [76,77], have been used for the treatment of SIBO in patients with functional bowel diseases.

An RCT compared the efficacy of 4-week courses of *Saccharomyces boulardii* at 250 mg bid and a multi-strain probiotic (*Bifidobacterium bifidum*, *Bifidobacterium longum*, *Bifidobacterium infantis*, and *Lactobacillus rhamnosus*) at 250 mg bid against placebo in the treatment of SIBO in patients with a diarrheal variant of IBS. Both drugs showed a positive effect. SIBO was eliminated in 40% of patients in the *Saccharomyces boulardii* group and in all 11 patients in the multi-strain probiotic group. However, SIBO was not eliminated in any of the 10 patients in the placebo group [78].

A multi-strain probiotic containing 25 billion active bacteria with 12 different strains (*Lactobacillus rhamnosus* at 6.0 billion CFU, *Bifidobacterium bifidum* at 5.0 billion CFU, *L. acidophilus* at 3.0 billion CFU, *L. casei* at 2.5 billion CFU, *L. plantarum* at 2.0 billion CFU, *L. salivarius* at 2.0 billion CFU, *B. longum* at 1.0 billion CFU, *Streptococcus thermophilus* at 1.0 billion CFU, *L. bulgaricus* at 1.0 billion CFU, *L. paracasei* at 0.5 billion CFU, *B. lactis* at 0.5 billion CFU, and *B. breve* at 0.5 billion CFU) eliminated SIBO in 33.3% of patients with diarrhea-predominant IBS in an uncontrolled study [79].

A probiotic containing *Bacillus subtilis* and *Enterococcus faecium* at 1500 mg/day for 4 weeks eliminated SIBO in 53% of patients with functional bowel disorders, and the probiotic was significantly more effective than placebo [80]. *Bifidobacterium infantis* M-63 at 1 × 10^9^ CFU/day for 3 months had no significant effect on SIBO [81].

#### 3.1.5. Other Interventions in the Treatment of SIBO in Functional Bowel Diseases

Drugs from other groups have also been assessed for the treatment of SIBO in functional bowel disease patients.

Herbal preparations administered for 4 weeks demonstrated a similar effect on the eradication of SIBO as rifaximin at 1200 mg/day (46% vs. 34%). Moreover, herbal preparations eliminated SIBO in 57% of patients who did not respond to rifaximin [82].

An RCT compared the efficacy of 4-week courses of prucalopride at 2 mg/day and a multi-strain probiotic (*Bifidobacterium bifidum*, *Bifidobacterium longum*, *Bifidobacterium infantis*, *Lactobacillus rhamnosus*) at 250 mg bid with placebo for the treatment of SIBO in patients with a constipation variant of IBS. Both drugs showed a positive effect. SIBO was eliminated in both patients in the prucalopride group and in all nine patients in the multi-strain probiotic group, but in none of the nine patients in the placebo group [83]. Prucalopride is a prokinetic laxative that accelerates intestinal peristalsis [84], the slowing down of which is one of the factors involved in the development of SIBO.

A palatable elemental diet for 2 weeks eliminated methane, hydrogen, and mixed SIBO in 58%, 100%, and 75% of cases, respectively; however, the study lacked a control group [85]. Lubiprostone is a laxative that stimulates the intestines to secrete chloride-rich fluid, increasing the volume of intestinal contents. The administration of lubiprostone at 24 mcg orally bid for 2 weeks eliminated SIBO in 41% of patients with chronic constipation [86]. Simethicone, an anti-foaming agent used to reduce bloating [87], and the herbal supplement curcumin [88] were not effective in treating SIBO.

#### 3.1.6. Drugs for the Treatment of SIBO in Functional Bowel Diseases: Conclusions

Rifaximin remains the most extensively studied drug for the treatment of SIBO in functional bowel disease. However, unlike many uncontrolled studies, placebo-controlled RCTs have demonstrated limited efficacy. This highlights the need for larger placebo-controlled RCTs. In comparative RCTs without placebo controls, some antibiotics (ciprofloxacin) showed a similar effect to rifaximin, whereas others were superior (metronidazole at 1500 mg/day) or inferior (metronidazole at 750 mg/day and chlortetracycline). Two antibiotics were shown to be superior to placebo in the treatment of SIBO in functional bowel disease in RCTs. However, the eradication rate in the study evaluating neomycin was too low, and the study on norfloxacin included too few participants. Drugs from other groups (probiotics, laxatives, herbal supplements, and an anti-foaming agent) showed variable effects in the treatment of SIBO.

### 3.2. Treatments for SIBO in Non-Functional Gut Diseases

SIBO is often detected in patients with Crohn’s disease [89,90], diverticular disease [91], lactase deficiency [92], short bowel syndrome [93], celiac disease [94], and other non-functional gut diseases [21] and significantly contributes to the digestive symptoms associated with these diseases.

#### 3.2.1. Treatment of SIBO in Crohn’s Disease

Rifaximin eliminated SIBO in patients with inactive Crohn’s disease more effectively than placebo in a small RCT (100% vs. 28.5%; 7 patients in each group); however, SIBO recurred in all patients at 1 month after the start of therapy (Table 5) [95]. An RCT showed that metronidazole and ciprofloxacin eliminated SIBO in Crohn’s disease patients with similar efficacies, and the effects were accompanied by reductions in the severity of bloating, stool softness, and abdominal pain [96]. Given the high recurrence rate of SIBO, patients should undergo testing for this condition periodically after successful eradication. The exact retesting regime should be established in future studies.

#### 3.2.2. Treatment of SIBO in Diverticular Disease

Rifaximin was more effective than placebo in relieving SIBO (83.3% vs. 8.3%) in patients with uncomplicated diverticular disease in a small RCT (12 patients in each group). Moreover, SIBO was eliminated in 78% of patients in the placebo group after treatment with rifaximin [97]. Rifaximin at 800 mg/day plus mesalazine at 2.4 g/day for 10 days, followed by mesalazine at 1.6 g/d for 8 weeks, eliminated SIBO in 98.8% of patients with acute uncomplicated diverticulitis, resulting in a significant improvement in symptoms [98].

#### 3.2.3. Treatment of SIBO in Lactose Intolerance

Rifaximin eliminated hydrogen SIBO in 32% of patients with lactose intolerance; however, the study lacked a control group [99].

#### 3.2.4. Treatment of SIBO in Short Bowel Syndrome

In the treatment of SIBO in children with short bowel syndrome, one antibiotic was sufficient in 56% of cases, two antibiotics had to be used in 36% of cases, and three antibiotics were used in the remaining 8%. Trimethoprim–sulfamethoxazole, metronidazole, amoxicillin/clavulanic acid, rifaximin, vancomycin, and gentamicin were used; however, the effectiveness of each agent was not reported. The average duration of therapy was 7–16 days [100].

#### 3.2.5. Treatment of SIBO in Celiac Disease

All 10 patients with SIBO and celiac disease were successfully treated with rifaximin at 800 mg/day for 1 week, which resulted in relief of symptoms; however, this study lacked a control group [101]. Rifaximin at 1200 mg daily for 10 days had no significant effect regarding the treatment of SIBO in patients with celiac disease and a poor response to a gluten-free diet compared with placebo in a small RCT (33.3% vs. 33.3%) [102].

#### 3.2.6. Treatment of SIBO in Chronic Intestinal Pseudo-Obstruction

Rifaximin at 400 mg tid for 4 weeks eradicated SIBO in 75% of patients with chronic intestinal pseudo-obstruction in an RCT. This rate was higher than that in the placebo group (25%). This is potentially due to the small number of participants (4 in each group), and this difference did not reach the limits of significance. After 8 weeks, SIBO recurred in all patients in the rifaximin group [103]. Given the high recurrence rate of SIBO, patients should undergo periodic testing for this condition after successful eradication. The exact retesting regime should be established in future studies.

**Table 5:** The efficacy of drugs in the treatment of SIBO in non-functional bowel diseases.

Disease	Drug	Dose	Design	SIBO Elimination Rate	Ref.
Crohn’s disease	Rifaximin	1200 mg for 7 days	pRCT	100% *	[95]
	Metronidazole	750 mg for 10 days	RCTwoP	86.7%	[96]
	Ciprofloxacin	1000 mg for 10 days	RCTwoP	100%	[96]
Uncomplicated diverticular disease	Rifaximin	1200 mg for 14 days	pRCT	83.3% *	[97]
Lactose intolerance	Rifaximin	1200 mg for 14 days	uCS	32%	[99]
Celiac disease	Rifaximin	800 mg for 7 days	uCS	100% *	[101]
Celiac disease resistant to a gluten-free diet	Rifaximin	1200 mg for 10 days	pRCT	33.3% *	[102]
Chronic intestinal pseudo-obstruction	Rifaximin	1200 mg for 28 days	pRCT	75% *	[103]

Note: * very small study; pRCT-RCT, with placebo; RCTwoP-RCT, without placebo; uCS, uncontrolled study. Studies with complex designs are not included.

## 4. Treatment of SIBO in Cirrhosis and Other Digestive Diseases

### 4.1. Treatment of SIBO in Cirrhosis

SIBO plays a special role in cirrhosis. Due to disruption of the intestinal, immune, and liver barriers, the development of SIBO leads to bacterial translocation and systemic inflammation. This induces systemic vasodilation, arterial hypotension, compensatory fluid retention, and hyperdynamic circulation. The latter aggravates the course of portal hypertension [104]. This explains why SIBO is associated with many complications of cirrhosis [11,105] and an unfavorable prognosis [12]. Thus, it is especially important to identify and treat this gut microbiota disorder in the context of this disease.

A blinded, randomized, placebo-controlled study showed that a probiotic containing *Saccharomyces boulardii* CNCM I-745 given at a dose of 250 mg twice daily for 3 months eliminated SIBO in 80% of decompensated cirrhosis patients (compared to 23% in the placebo group; Table 6). This effect was accompanied by a decrease in the severity of hepatic encephalopathy, ascites, hyperbilirubinemia, thrombocytopenia, hypoalbuminemia, hemodynamic changes, biomarkers of bacterial translocation, endothelial dysfunction, and systemic inflammation [13].

Rifaximin at 600 mg/day for 1 week eliminated SIBO in 76% of patients with cirrhosis and minimal hepatic encephalopathy. This effect was accompanied by a more significant decrease in hyperammonemia and the severity of minimal hepatic encephalopathy than that in individuals without SIBO at inclusion [106]. Rifaximin at 1200 mg/day eliminated hydrogen SIBO in 66.7% and methane SIBO in 20% of patients with cirrhosis and covert hepatic encephalopathy in a small study (including only eight patients) [107]. Rifaximin at 1200 mg/day for 1 week eliminated SIBO in 94% (16/17) of patients with cirrhosis and hepatic encephalopathy. This effect was accompanied by a significant improvement in mental function [108]. It should be noted that there were no control groups in these studies.

The administration of the multi-strain probiotic VSL#3 for 3 months eliminated SIBO in 57.6% of patients with cirrhosis and without overt hepatic encephalopathy, and this rate was significantly higher compared with that reported for the control group (19.2%) [109].

### 4.2. Treatment of SIBO in Other Digestive Diseases

When administered for 2 months, a probiotic containing Bifidobacterium eliminated SIBO in 80% of patients with idiopathic halitosis, and this effect was almost always accompanied by normalization of oral odor. There was no control group [110].

A meta-analysis of rifaximin treatment showed that this antibiotic eliminated SIBO in 76% of cases of chronic pancreatitis [111]. The administration of rifaximin at 1200 mg for 7 consecutive days each month for 3 months cured SIBO in all 12 patients with chronic pancreatitis, and this effect was accompanied by a reduction in diarrhea [112].

Bismuth-based quadruple eradication therapy for Helicobacter pylori infection eliminated hydrogen and methane SIBO in 66.7% and 76.9% of patients with this infection, respectively [113]. Eradication therapy consisting of pantoprazole (2 × 40 mg), amoxicillin (2 × 1000 mg), and metronidazole (2 × 500 mg) for 10 days eliminated SIBO in 60.0% of patients with Helicobacter infection. The substitution of metronidazole for rifaximin (1200 mg) resulted in a non-significant increase in therapeutic efficacy to 75%. The efficacy of eradication therapy was similar in both groups [114].

Ursodeoxycholic acid at 100 mg tid for 2 months eliminated hydrogen SIBO in 75% of patients with functional dyspepsia (placebo was effective in none). However, regarding methane SIBO, this drug had an identical effect to placebo. The number of patients studied was very small (less than 10 patients in each group) [115].

## 5. Treatment of Iatrogenic SIBO

### 5.1. Treatment of Drug-Induced SIBO

SIBO is considered as drug-induced if its development is associated with medication use. Rifaximin at a dose of 1200 mg/day for 14 days eliminated 68% of cases of hydrogen SIBO and 53% of cases of methane SIBO that developed after long-term use of proton pump inhibitors [116].

### 5.2. Treatment of Postoperative SIBO

SIBO is considered postoperative if it develops after surgery. A probiotic regimen containing *Lactobacillus acidophilus*, *Bifidobacterium lactis*, *Lactobacillus rhamnosus*, *Bifidobacterium longum*, *Lactobacillus plantarum*, *Bifidobacterium bifidum*, and *Lactobacillus gasseri* at a dose of 50 billion CFU/day for 8 weeks and another probiotic regimen based on Lactobacillus acidophilus and *Bifidobacterium lactis* (5 billion CFU/strain) for 90 days had no significant effect on SIBO development in patients after Roux-en-Y gastric bypass bariatric surgery [117,118].

Metronidazole at a dose of 750–1000 mg/day for 7 days every month for 3 months eliminated postoperative SIBO in 58% of cases. The addition of gentamicin 80 mg/day to this regimen did not significantly alter its efficacy [119].

Rifaximin at 1200 mg/day for 10 days and metronidazole at 1500 mg/day were not effective in the treatment of SIBO as a complication of gastrectomy, with eradication rates of 5.4% and 14.3%, respectively [120]. Rifaximin at 1200 mg/day for 10 days eliminated SIBO in 33.3% of patients undergoing surgery for colorectal cancer. This effect was accompanied by a decrease in the severity of digestive symptoms, which remained unchanged in the subgroup of patients who did not respond to this drug [121].

## 6. Treatment of SIBO in Other Diseases

SIBO is often detected in patients with diseases of organs not related to the digestive system [21].

### 6.1. Treatment of SIBO in Systemic Sclerosis

A meta-analysis of nine studies on the effectiveness of antibiotics in the treatment of SIBO in systemic sclerosis showed that these drugs eliminate SIBO in 56% (95% CI: 48–65%) of cases. Rifaximin was more effective than other antibiotics (78% [95% CI: 65–88] vs. 45% [95% CI: 32–58]). No studies compared an antibiotic group with a placebo or an untreated control group. Most studies did not report the development of significant adverse effects. Only one case of pseudomembranous colitis in response to treatment of SIBO was described [122].

García Collinot et al. compared metronidazole (500 mg bid for 7 days), *Saccharomyces boulardii* (200 mg bid for 7 days), and their combination (in this case, *Saccharomyces boulardii* was taken for 14 days) in the eradication of SIBO in systemic sclerosis. This therapy was repeated 1 month after its start. After the first course of *Saccharomyces boulardii* therapy, SIBO was eradicated in 25% of patients. After the second course, this rate increased to 33%. These values were 30% and 25% for metronidazole and 48% and 55% for the combination therapy, respectively [123].

### 6.2. Treatment of SIBO in Cystic Fibrosis

Oral administration of ciprofloxacin eliminates SIBO in cystic fibrosis more effectively than intravenous amikacin and ceftazidime (90% vs. 44%) [124]. A small RCT showed that rifaximin eliminates SIBO in cystic fibrosis better than placebo (9/10 vs. 2/6) [125]. The CFTR modulators had no significant effect on SIBO in cystic fibrosis [126].

### 6.3. Treatment of SIBO in Other Non-Digestive Diseases

The results of treatment of SIBO in various non-digestive diseases are detailed in Table 7 and Table 8.

## 7. Summary, Limitations, and Perspectives

The most extensively studied drug for the treatment of SIBO in various diseases is rifaximin. However, most of the studies have lacked a control group. Increasing the dose of this drug improved its effectiveness. Small RCTs assessing the effects of rifaximin in SIBO have reported conflicting results, and a meta-analysis showed no effect. A large RCT is required to verify the positive effect of rifaximin in the treatment of SIBO. Neomycin and norfloxacin have also shown efficacy in the treatment of SIBO in single RCTs. Other antibiotics have also shown positive effects for the treatment of SIBO, but only in uncontrolled studies or in comparison with rifaximin or each other. Various probiotics have been tested in the treatment of SIBO in the context of various diseases, including VSL#3 and *Saccharomyces boulardii* CNCM I-745, both of which showed positive effects in RCTs.

Rifaximin demonstrated an excellent safety profile with minimal adverse effects in all studies. Similar data were obtained for probiotics. The use of antibiotics other than rifaximin resulted in more frequent adverse effects.

Therefore, the main limitation for a guideline on SIBO treatment is that most of the data are derived from uncontrolled studies, and most of the RCTs conducted included limited numbers of participants. Another limitation is that different studies used varying breath test substrates, cut-off values, and testing protocols, which may affect SIBO detection rates and complicate cross-study comparisons.

It appears promising to investigate the effectiveness of different diets, exercises, prebiotics, postbiotics, new antibiotics, and fecal transplantation for the treatment of SIBO under specific conditions. A palatable elemental diet showed good effects on SIBO in functional bowel diseases in an uncontrolled study [85]. In a recent RCT without a placebo control, a low FODMAP diet for 4 weeks showed a similar SIBO eradication rate in IBS patients as rifaximin 1200 mg for 14 days (50.0% vs. 63.6%) [137]. The same diet eliminated SIBO in 80.9% of patients with halitosis, and this effect was accompanied by an improvement in symptoms of the underlying disease in an uncontrolled retrospective study [138].

Fecal microbiota transplantation reduced intestinal hydrogen production in patients with SIBO and Parkinson’s [139] and functional bowel diseases [140]. However, SIBO eradication rates were not reported in these studies [139,140]. This procedure eliminated SIBO in 71% of patients with chronic intestinal pseudo-obstruction in a small uncontrolled study [141].

Currently, several RCTs have been conducted to assess SIBO treatments (Table 9).

## 8. Conclusions

When selecting SIBO treatment regimens, those that have demonstrated efficacy for the specific underlying condition should be preferred, particularly those shown to be efficacious in RCTs, although such data remain limited. In the absence of RCT evidence, regimens that have demonstrated the highest SIBO eradication rates in uncontrolled studies may be considered. The low level of evidence should be kept in mind in this case. Large placebo-controlled RCTs are necessary to accurately evaluate the efficacy of drugs and other interventions for the treatment of SIBO in specific clinical conditions.

## Figures and Tables

**Table 1:** Comparison of the efficacy of different rifaximin doses in the treatment of SIBO according to a recent meta-analysis [32].

Rifaximin Dose, mg/d	Eradication Rate for SIBO, %
600	45
800	49
1200	64
1600	80

**Table 4:** The efficacy of rifaximin in the treatment of SIBO in functional bowel diseases based on randomized trials.

Dose	Comparator	SIBO Elimination Rate for Rifaximin and Comparator	Conclusion: Rifaximin vs. Comparator	Ref.
RCT without Placebo				
1200 mg for 7 days	Chlortetracycline 1000 mg for 7 days	70% vs. 27%	Superior	[67]
	Metronidazole 750 mg for 7 days	63% vs. 44%	Superior	[68]
800 mg for 10 days	Metronidazole 1500 mg for 10 days	59% vs. 79%	Inferior	[69]
	Ciprofloxacin 1000 mg for 10 days	59% vs. 54%	Similar	[69]
RCT with Placebo				
1100 mg for 14 days	Placebo	13% vs. 18% *	Similar	[74]
1650 mg for 10 days	Placebo	20% vs. 14% **	Similar	[75]

Note: * Hydrogen SIBO, ** in children.

**Table 6:** The efficacy of different drugs in the treatment of SIBO in the context of cirrhosis.

Drug	Dose	Design	SIBO Elimination Rate	Ref.
*Saccharomyces boulardii* CNCM I-745	500 mg for 3 months	pRCT	80%	[13]
Rifaximin	600 mg for 7 days	uCS	76%	[106]
	1200 mg	uCS	67% *	[107]
	1200 mg for 7 days	uCS	94%	[108]
VSL#3	3 × 10^8^ cells for 3 months	RCTwoP	57.6%	[109]

Note: * very small study; pRCT-RCT, with placebo; RCTwoP-RCT, without placebo; uCS, uncontrolled study.

**Table 7:** Results of the treatment of SIBO in various non-digestive diseases.

Underlying Disease	Drug	Dose, mg/Day	Duration, Days	SIBO Eradication Rate, %	RCT	Reference
Cystic fibrosis	Rifaximin	1200	14	90	Yes	[125]
Hypothyroidism	Rifaximin	1200	7	70	No	[127]
Hypothyroidism in early pregnancy	*Bifidobacterium infantis*, *Lactobacillus acidophilus*, *Enterococcus faecalis*, and *Bacillus cereus*	4500	21	71	No	[128]
Acromegaly	Rifaximin	1200	10	53	No	[129]
Systemic sclerosis	Rifaximin	1200	10	73	No	[130,131,132]
Parkinson’s disease	Rifaximin	1200	7	78 *	No	[133]
HIV-associated autonomic neuropathies	Pyridostigmine	90	56	67	No	[134]
Rosacea	Rifaximin	1200	10	78	No	[135]
	Rifaximin	1200	10	88	Yes	[136]

Note: * SIBO recurred in 43% (6/14) of patients 6 months after the end of therapy.

**Table 8:** Effects of SIBO eradication in various diseases.

Underlying Disease	Effects of SIBO Eradication	Reference
Cystic fibrosis	Improved fat digestion and absorption	[124]
Hypothyroidism	Decreased the severity of abdominal discomfort, bloating and flatulence; no effect on thyroid hormone serum levels	[127]
Acromegaly	Improved digestive symptoms	[129]
Systemic sclerosis	Improved digestive symptoms	[130,131,132]
Parkinson’s disease	Improved motor fluctuations without affecting the pharmacokinetics of levodopa	[133]
Rosacea	Partial or complete regression of skin symptoms	[135,136]

**Table 9:** Current RCTs of SIBO treatments.

Study	Disease	Intervention
NCT07451171	Functional bowel diseases	Metronidazole 20–30 mg/kg/day vs. trimethoprim–sulfamethoxazole 8–10 mg/kg/day + metronidazole 20–30 mg/kg/day vs. amoxicillin–clavulanate 20–40 kg/day for 14 days
NCT07424313		Low FODMAP diet vs. healthy eating diet
NCT06317441		Probiotic vs. placebo
NCT06721884		*Artemisia annua* leaf vs. placebo
NCT07426705	IBS	Probiotic + rifaximin vs. placebo + rifaximin

## Data Availability

No new data were created or analyzed in this study.

## Abbreviations

The following abbreviations are used in this manuscript:

IBS	Irritable bowel syndrome
RCT	Randomized controlled trial
SIBO	Small Intestinal Bacterial Overgrowth
